# Microbial recovery of rare earth elements from various waste sources: a mini review with emphasis on microalgae

**DOI:** 10.1007/s11274-024-03974-4

**Published:** 2024-05-04

**Authors:** Milada Vítová, Dana Mezricky

**Affiliations:** 1 Department of Phycology, Institute of Botany of the Czech Academy of Sciences, Třeboň, Czechia; 2 Institute of Medical and Pharmaceutical Biotechnology, IMC Krems, Krems, Austria

**Keywords:** Rare earth elements, Recovery, Microorganisms, Algae, Bioleaching, Bioaccumulation

## Abstract

Rare Earth Elements (REEs) are indispensable in contemporary technologies, influencing various aspects of our daily lives and environmental solutions. The escalating demand for REEs has led to increased exploitation, resulting in the generation of diverse REE-bearing solid and liquid wastes. Recognizing the potential of these wastes as secondary sources of REEs, researchers are exploring microbial solutions for their recovery. This mini review provides insights into the utilization of microorganisms, with a particular focus on microalgae, for recovering REEs from sources such as ores, electronic waste, and industrial effluents. The review outlines the principles and distinctions of bioleaching, biosorption, and bioaccumulation, offering a comparative analysis of their potential and limitations. Specific examples of microorganisms demonstrating efficacy in REE recovery are highlighted, accompanied by successful methods, including advanced techniques for enhancing microbial strains to achieve higher REE recovery. Moreover, the review explores the environmental implications of bio-recovery, discussing the potential of these methods to mitigate REE pollution. By emphasizing microalgae as promising biotechnological candidates for REE recovery, this mini review not only presents current advances but also illuminates prospects in sustainable REE resource management and environmental remediation.

## Introduction

Rare earth elements (REEs) are groups of 17 elements in the periodic table including yttrium, scandium and 15 lanthanides. Despite their name, REEs are not actually rare in terms of their abundance in the Earth’s crust. However, they are often dispersed in low concentrations, making their extraction and separation complex and expensive. Due to their importance in modern technologies, there has been growing concern about the supply and sustainability of REEs. They are integral to many aspects of human lives, from the devices we use to the technologies that help address environmental challenges. They are critical in a variety of products including permanent magnets in electric motors or wind turbines, catalysts, lighting, electronics, advanced weapons systems or clean energy technologies like solar panels and rechargeable batteries (Balaram 2019). China has historically dominated the production of rare earths, which has led to concerns about supply chain security. For example, US imported 80% of REEs from China in 2022 and EU imports up to 98% (U.S. Geological Survey 2023; European Commission 2023). Efforts are being made in various countries to diversify rare earth production and recycling methods to reduce dependency on a single source (Brown et al. 2023).

REEs are primarily mined and extracted from ore sources like monazite, bastnasite, or xenotime by heating in acids and solvents (Congressional Research Service 2020). Solid and liquid REE-bearing wastes are generated in various industries (Omodara et al. 2019). Notably, there has been an explosive increase in waste electric and electronic equipment (WEEE). The WEEE were estimated at over 52 million metric tons by 2021 and are considered as secondary sources of REEs (Isildar et al. 2019). Conventional physicochemical methods of REE recovery (e.g., solvent extraction, ion exchange, coprecipitation, and crystallization) are energy-intensive and often cause further pollution. Low-cost and eco-friendly technologies including biosorbents, bio-electrochemical systems, bioleaching, and biomineralization, have become alternatives in the recovery of REEs (Yu et al. 2020). Microorganisms, including bacteria, algae, fungi, yeast and archaea, have been found to play a significant role in the natural cycling of REEs and researchers are exploring the potential of harnessing these microorganisms for the purpose of recovering REEs from various sources (Jalali and Lebeau 2021). However, a relatively low recovery rate and selectivity severely hinder their large-scale applications. Nevertheless, analyses in terms of economic perspectives indicate that REE bio-recovery from waste materials may be a cost-effective approach. This focus was expanded to exploit novel strain resources, genetic engineering of strains and other strategies to improve bio-recovery efficiency (Jung et al. 2023).

**Fig. 1:**
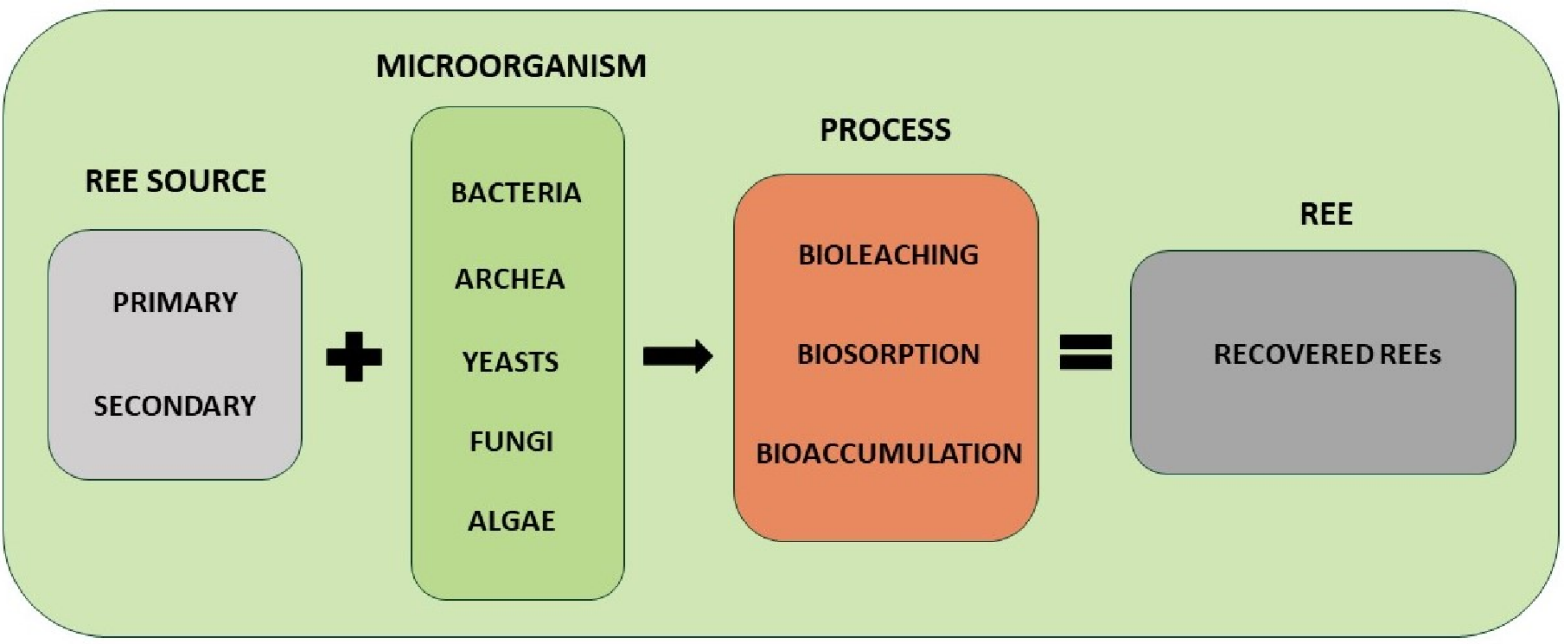
Scheme of bio-recovery of rare earth elements

**Fig. 2:**
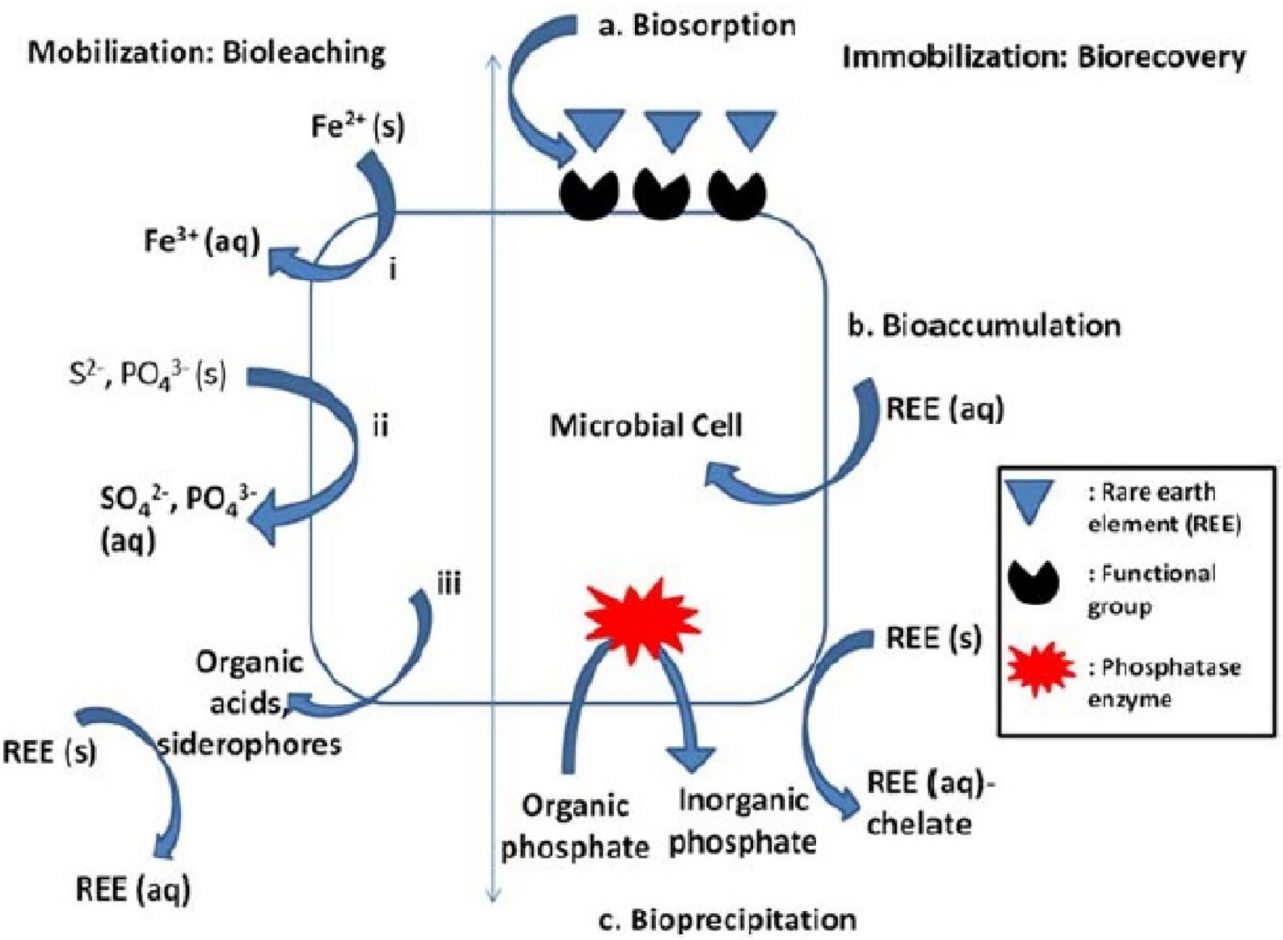
Scheme of the mechanisms of uptake and bio-recovery of rare earth elements. Solid to aqueous phase mobilization occurs through (i) redoxolysis, (ii) acidolysis and (iii) complexolysis methods. REEs can either (a) bind with functional groups on microbial surface—biosorption, (b) accumulate inside the cell through channels—bioaccumulation, or (c) get precipitated with inorganic phosphate liberated through phosphatase enzymes—bioprecipitation (Lhamo and Mahanty 2022)

The bio-recovery of REEs include particularly bioleaching, biosorption or bioaccumulation (Fig. 1). Bioleaching is based on dissolving REEs from the mineral matrix with organic acids produced by some bacteria and archaea (Dev et al. 2020). Biosorption is a passive, non-metabolic process where REEs are bound to functional groups (such as carboxyl, hydroxyl, and phosphate groups) on the cell walls of both living and dead cells (Giese 2020). In contrast, bioaccumulation is an active process where metals must enter the cell and accumulate inside. Metal uptake is only possible in living cells (Zabochnicka-Świątek and Krzywonos 2014). The mechanisms of uptake and bio-recovery of REEs on the cellular level are schematically summarized in the Fig. 2.

## Mechanisms of bio-recovery

### Bioleaching

Bioleaching is a process by which microorganisms, usually bacteria or fungi, are used to extract metals from ores, minerals, or secondary sources (Adentuji et al. 2023; Devi and Ganesh 2023). This method is an alternative to traditional chemical or physical processes for metal extraction. Bioleaching is particularly effective for the recovery of metals from low-grade ores or complex mineral sources that are not easily processed using conventional methods.

The bioleaching process generally involves the following steps: (a) Attachment and colonization: microorganisms attach themselves to mineral surfaces; (b) Metabolic activity: Microorganisms release organic acids, enzymes, and other metabolites, promoting the dissolution of minerals and leaching of metals; (c) Recovery: Dissolved metals can be recovered through various methods, such as precipitation, solvent extraction, ion exchange or bio-methods.

Bioleaching has several advantages over traditional methods, including lower energy consumption, reduced environmental impact, and the ability to process ores using methods that are not economically viable using conventional techniques (Pollmann et al. 2018). It is used for the extraction of various metals including REEs. However, the success of bioleaching depends on factors such as the type of microorganisms used, characteristics of the ore, and the environmental conditions of the bioleaching operation (temperature, pH, pulp density, particle size, medium composition, aeration and stirring) (Owusu-Fordjour and Yang 2023).

## Biosorption

Biosorption is defined as the transport of toxic metals from aqueous solutions to the surface of dead or living biomass (Jais et al. 2017). Research is focused on removal of toxic metals or metalloids by biomass of different origins. In contrast to physical and chemical methods, biosorption is quick, reversible, inexpensive, and environmentally friendly. Apart from plants, algae are well studied naturally inspired biosorbents that offer various advantages (Goswami et al. 2022). These are mainly: easy production, cultivation under a wide range of growth conditions and high metal binding capacity (Ramírez-Calderón et al. 2020). Cell walls play a main role in the biosorption of metals, exposing binding sites for metal ions. The functional groups (OH^−^, SO_4_^2−^, NH_2_ etc.) of polysaccharides, lipids, and proteins of algal cell walls (these differ in red, brown, or green algae) act as binding sites (Cheng et al. 2019; Giese 2020). The most studied microalga for biosorption is the green alga *Chlamydomonas reinhardtii* due to its unicellular nature, rapid growth, and ability to adapt to various environments (Mantzorou et al. 2018). Its cell wall structure and surface characteristics make it suitable for adsorbing heavy metals and other pollutants, making it a promising candidate for biosorption studies. In addition, its relatively simple genetic arrangement facilitates genetic engineering for enhanced biosorption capabilities.

There are several reports that show that living algal biomass can be effectively used for biosorption, but mostly inactive biomass and non-living algae have been used for this purpose, including carbonized *Parachlorella* biomass used for recovery of REEs from clay minerals (Ponou et al. 2014). According to Kumar et al. (2009), five green marine macroalgae, namely *Cladophora fasicularis*, *Ulva lactuca, Chaetomorpha* sp, *Caulerpa sertularioides*, *Valoniopsis pachynema* can accumulate significant amounts of heavy metals.

## Bioaccumulation

Bioaccumulation has been referred to as a two-phase mechanism. The first phase represents passive metal binding to the cell wall. A second phase, metal uptake into the cell, is only possible in living cells (Zabochnicka-Świątek and Krzywonos 2014). Microalgae use various strategies to maintain intracellular metal concentrations at optimal levels and to prevent the entry of non-essential metals in order to maintain inner metal homeostasis (Torres et al. 2008). These mechanisms include metal exclusion or metallic efflux systems and intracellular accumulation. Intracellular accumulation of metals represents an important mechanism for metal tolerance and detoxification (Sriprang and Murooka 2007). Microalgae can produce chelators that are able to complex with metals. These complexes are then localized in the cell to avoid the toxic effects of metals (Nowicka 2022). Metallothioneins (MTs) and phytochelatins (PCs) are the most powerful compounds in metal detoxification, probably having independent functions (Shukla et al. 2016). MTs are metal binding proteins controlling physiological intracellular metal levels (Gaur and Rai 2001). Molecules of MTs possess a number of sulfhydryl groups that allow the binding of metals (Joshi et al. 2016). PCs are short polypeptides found in higher plants, algae (including cyanobacteria), yeasts and nematodes (Wang et al. 2017), playing a role in metal detoxification (Shukla et al. 2016). They were proven to be synthesized and used in algae for detoxification (Balzano et al. 2020). Metals bound to PC are stored in vacuoles, playing a key role in the metal detoxification of the cytoplasm (Joshi et al. 2016; Sriprang and Murooka 2007). Several studies have shown that REEs can accumulate in chloroplasts of algae (Guo et al. 2000; Shen et al. 2002, 2003). Similarly, the REEs Nd and Ce preferentially accumulated in chloroplasts in the green alga *Desmodesmus quadricauda* while La and Gd were found in the cytoplasm (Řezanka et al. 2016).

## Bio-recovery by microalgae

Common microalgae that can be used for metal recovery or wastewater treatment cover a wide spectrum of green, red and brown algae and cyanobacteria. Thanks to their ability to fix CO_2_ and grow phototrophically they are promising cell factories to produce bioenergy and high-value products with potential for a circular economy (Anyaoha et al. 2024; Goswami et al. 2023). Except for photoautotrophy, there are several types of algae able to grow under chemoheterotrophic or mixotrophic metabolic regimes, which is beneficial in the use of industrial wastewaters containing high organic loads. The most studied strains from this point of view are the green alga *Chlorella vulgaris*, and the cyanobacterium *Arthrospira platensis*, cultivated on a wide range of wastewaters of different origins (Wollmann et al. 2019).

Microalgae specialized for growth on harsh habitats (so called extremophiles) are potential candidates for biotechnologies. Strains isolated from such places can grow under severe conditions needed for metal recovery (Malavasi et al. 2020). Such conditions include very low or very high pH (pH2 or pH11), extreme temperatures (< 10 °C or > 40 °C), high organic doses, high salt, or high metal levels. Extremophiles consist of two categories—extremotolerant microorganisms that can survive under extreme conditions but grow optimally under normal conditions and those that need extreme conditions for their metabolic activity (Rampelotto 2013; Varshney et al. 2015).

One of the promising extremophiles with a strong biotechnological potential is the unicellular red alga *Galdieria sulphuraria* (Čížková et al. 2019b). Strains of this Rhodophyta can grow not only phototrophically but also mixotrophically and heterotrophically on 27 different sugars and sugar alcohols; this is unique among microalgae (Gross and Schnarrenberger 1995; Náhlík et al. 2021). *G. sulphuraria* withstands highly acidic environments, up to pH 1.8, and temperatures up to 56 °C (Merola et al. 1981; Selvaratnam et al. 2014). Metabolic diversity, combined with the production of high value phycobiliprotein phycocyanin, makes *G. sulphuraria* a very promising tool for biotechnology (Wan et al. 2016). The next promising acidophilic strain is the green alga *Chlamydomonas acidophila* isolated from an acidic river in a mining area with a very low pH, and able to grow mixotrophically on glucose, glycerol, and starch (Cuaresma et al. 2011). It produces antioxidants such as the carotenoid lutein (Garbayo et al. 2008).

The advantage of using microalgae for bio-recovery lies mainly in their simple cell structures, easy access to CO_2_ and nutrients and the ability to grow in extreme environments (Leong and Chang 2020). As a result, they are more efficient in converting energy into biomass (Priyadarshani et al. 2012). Several species of microalgae are already known to be powerful accumulators of toxic metals (e.g., *Scenedesmus subspicatus, Chlamydomonas sp., Cyclotella cryptica, Phaeodactylum tricornutum, Porphyridium purpureum, Phormidium ambiguum, Pseudochlorococcum typicum, Chlorella kessleri*, *Chlorella vulgaris*, *Phormidium* sp*., Rhizoclonium hookeri, Spirulina* sp., etc.) (Guleri et al. 2020; Schmitt et al. 2001; Shanab et al. 2012). Their success strongly depends on conditions and species used. Moreover, the microalgal biomass can be further reused for other applications such as biofuel production, aquaculture and animal feed, fertilizers, or for the biosynthesis of bioactive compounds such as vitamins and pigments (Goswani et al. 2022; Brar et al. 2017; Schnurr and Allen 2015).

## Bio-recovery of REEs

### Bacteria, yeast, and fungi

Bio-recovery or bioleaching of REEs using bacteria, yeast, and fungi is an innovative and environmentally friendly approach to extract these valuable materials from ores (namely monazite, bastnasite, or apatite) or various industry wastes (Vo et al. 2024). REEs occur in various forms as phosphates, sulphates, or silicates in these primary resources. Secondary REE resources typically comprise wastes like phosphogypsum, red mud, coal-related resources, and WEEE (Fathollahzadeh et al. 2019; Shi et al. 2023). Traditional extraction methods often involve the use of harsh chemicals and can have significant environmental impacts. Bio-recovery, on the other hand, harnesses the metabolic capabilities of microorganisms to selectively leach and recover REEs (Danouche et al. 2024). Microorganisms active in bioleaching can be both autotrophic and heterotrophic.

Certain bacteria, such as acidophilic bacteria, are well-suited for bioleaching. They thrive in acidic environments and produce organic acids that help dissolve minerals containing REEs. Bacterial strains like *Acidithiobacillus ferrooxidans* and *Acidithiobacillus thiooxidans* were studied for their ability to solubilize REEs from ores (Hong et al. 2023; Hosseini et al. 2022; Tayar et al. 2022). Bioleaching by bacteria can be followed by accumulation of REEs by algae *Euglena* sp. (EugVP) and *Chlamydomonas* sp. (ChlSG) (García-Balboa et al. 2022).

Some yeast species were found to play a role in bioleaching processes. Yeasts like *Yarrowia lipolytica* have demonstrated the ability to extract REEs from ores or electronic waste (Ferreira et al. 2022; Shen et al. 2023). Yeasts often produce organic acids and excrete metabolites that facilitate the dissolution of minerals and enhance the availability of REEs.

Fungi are also employed in bioleaching due to their ability to produce organic acids and enzymes that can break down mineral structures. Fungal species like *Aspergillus niger* or *Penicillium* spp. were investigated for their potential to extract REEs and were reported as being the two most common chemoautotrophs used for bioleaching (Owusu-Fordjour and Yang 2023; Zhou et al. 2024). Microorganisms efficient in the bioleaching of REEs are summarized in Table 1.

**Table 1:** Bacteria, yeasts and fungi efficient in REE recovery

REE	Microorganism	Mechanism	Conditions	References
La	*Magnetospirillum magneticum* *Saccharomyces cerevisiae*	Biosorption Biosorption	batch, 4d, 30 °CC, pH6 batch, 30 °CC, pH4	Mohammadi et al. (2022) Di Caprio et al. (2016)
Ce, Dy, Er, Eu, Gd, Ho, La, Lu, Nd, Pr, Sm, Tb, Tm	*Acidophilium multivorum* *Leptospidillum ferriphilum*	Bioleaching Bioleaching	batch, 15d, 30 °CC, pH3 batch, 15d, 30 °CC, pH3	García-Balboa et al. (2022)
La, Nd, Ce; REE ore	*Yarrowia lipolytica* (IM-UFRJ 50678) *Yarrowia lipolytica*	Bioleaching Bioleaching	batch, 45 min, 50 °CC batch, pH6	Ferreira et al. (2022) Shen et al. (2023)
Heavy REEs	*Bacillus subtilis* *Leinsingeria methylohalidivorans*	Biosorption Biosorption	batch, 2–4d, 30 °CC batch, 2d, 20 °CC	Breuker et al. (2020) Takahashi et al. (2005)
La, Sm	*Bacillus subtilis*-alginate *Bacillus subtilis*	Biosorption Biosorption	batch, 1 h, 30 °CC batch, 20 min, 30 °CC, pH3	Coimbra et al. (2019) Giese and Jordao (2019)
Gd, Dy, Yb, Lu	*Saccharomyces cerevisiae*	Biosorption	batch, 2–4d, 30 °CC	Breuker et al. (2020)
La, Nd, Dy, Yb	*Pichia naganishii*	Biosorption	batch, 2–4d, 30 °CC	Breuker et al. (2020)
Preference for: Gd	*Pichia* sp*.*	Biosorption	batch, 2–4d, 30 °CC	Breuker et al. (2020)
Preference for: Ce, Nd, Gd, Dy	*Catenulostroma chromoblastomyces*	Biosorption	batch, 2–4d, 30 °CC	Breuker et al. (2020)
Preference for: Gd, Yb, Lu	*Pezicomycotina* sp*.*	Biosorption	batch, 10d, 30 °CC	Breuker et al. (2020)
Preference for: Nd, Gd, Gy, Lu	*Fusarium* sp.	Biosorption	batch, 2–4 d, 30 °CC	Breuker et al. (2020)
heavy REEs	*Escherichia coli*	Biosorption	batch, 30 min, 37 °CC	Takahashi et al. (2005) Park et al. (2020)
La, Eu, Yb	*Pseudomonas aeruginosa*	Biosorption	batch, 30 °CC, pH5	Texier et al. (1999)
Nd	*Kluyveromyces marxianus* *Candida colliculosa* *Debaromyces hansenii* *Saccharomyces cerevisiae*	Biosorption Biosorption Biosorption Biosorption	batch, pH1 batch, pH1 batch, pH1 batch, pH1	Vlachou et al. (2009)
Ce; REEs from industry wastes	*Aspergillus niger* *Aspergillus flavus* *Bacillus licheniformis*	Biosorption; Bioleaching Bioleaching Bioleaching Bioleaching	batch, 10d, 30 °CC, pH5 batch, 3d, 28 °CC, pH2 batch, 3d, 28 °CC, pH2 batch, 25 °CC, pH2 semicontinuous, 4d, 30 °CC, pH5.3	Cheng et al. (2022) Sallam et al. (2014) Barnett et al. (2020) Bahaloo-Horeh and Mousavi (2022) Shen et al. (2023) Ma et al. (2023) Castro et al. (2023b)
La, Ce	*Agrobacterium* sp. HN1	Biosorption	batch, 2 h, 30 °CC, pH6.8	Xu et al. (2011)
Sc, La, Sm, Y	*Candida utilis*	Biosorption	batch, pH5	Korenevsky et al. (1999)
Eu	*Bacillus thuringiensis*	Biosorption	batch, 2d, pH8	Pan et al. (2017)
Tb	*Caulobacter crescentum*	Biosorption	batch, 20 min, 30 °CC, pH6	Park et al. (2016)
REEs	*Arthrobacter niicotianae*	Bioleaching	batch, 30 min	Park et al. (2020)
REE ore	*Aspergillus niger* *Acidithiobacillus ferrooxidans* *Gluconobacter oxydans*	Bioleaching Bioleaching Bioleaching	batch, 7d, 38 °CC, pH5 batch, 60d, 30 °CC, pH3 batch, 5d, 30 °CC, pH3	Zhou et al. (2024) Fatollahzadeh et al. (2018b) Tian et al. (2022) Gao et al. (2023)
REEs from phosphogypsum; gold mine	*Acidithiobacillus thioxidans*	Bioleaching	batch, 8d, 30 °CC, pH3	Tayar et al. (2022) Hong et al. (2023) Hosseini et al. (2022)
REEs from monazite	*Enterobacter aerogenes* *Penicillium* sp*.* *Pantoea agglomerans* *Pseudomonas putida* *Burkholderia thailandensis*	Bioleaching Bioleaching Bioleaching Bioleaching Bioleaching	batch, 18d, 30 °CC batch, 8d, 30 °CC batch, 14d, 37 °CC batch, 14d, 37 °CC batch, 21d, 30 °CC, pH5.3	Fathollahzadeh et al. (2018a) Corbett et al. (2018) Castro et al. (2023a)
REEs from fly ash	*Candida bombicola* *Phanerochaete chrysosporium* *Cryptococcus curvatus*	Bioleaching Bioleaching Bioleaching	batch, 3d, 28 °CC, pH5.5 batch, 3d, 28 °CC, pH5.5 batch, 3d, 28 °CC, pH5.5	Park and Liang (2019)

Benefits of bio-recovery/bioleaching include reduced environmental impact, lower energy consumption, and often, a higher selectivity for target elements compared to conventional methods. Harnessing the biological capabilities of bacteria, yeast, and fungi for the bio-recovery of REEs holds great promise for future sustainable and eco-friendly mining practices. However, challenges such as the slow kinetics of the process and the need for optimization remain areas of ongoing research.

## Algae

Biosorption of REEs by algae was described, for example, in the seaweed *Sargassum* sp., where biomass quickly and efficiently acquired Eu, Gd, La, Nd, Pr and Sm (Oliveira and Garcia 2009; Oliveira et al. 2011, 2012). Similar results were obtained with other species of brown seaweed, such as *Sargassum polycystum* (Diniz and Volesky 2005, 2006) *Sargassum hemiphylum, Ulva pertusa*, *Schizymenia dubyi* (Kano 2013) and *Turbinaria conoides* (Vijayaraghavan et al. 2010, 2011). *Gracilaria gracilis* was able to efficiently accumulate, in 70% yield, low concentrations (0.5 mg/l) of REEs (Y, Ce, Nd, Eu and La) occurring in wastewater . The ability of *G. gracilis* to uptake REEs from such low concentrations overcomes one of the greatest difficulties in recycling these elements so far (Jacinto et al. 2018). A promising candidate for the selective recovery of Sc and REEs from the aquatic environment is the seaweed *Posidonia oceanica* (Ramasamy et al. 2019). Macroalga *Ulva* sp. was used for recovery of REEs from industrial wastewaters (Manikandan and Lens 2022; Viana et al. 2023).

Also, some microalgae, such as *Chlorella* spp. and *Nannochloropsis* spp. or cyanobacteria *Microcystis* spp. were shown to be active biosorbents of REEs (La^3+^ and Ce^3+^) (Richards and Mullins 2013; Zhou et al. 2004). The ability to accumulate REEs has also been demonstrated in the red alga *G. sulphuraria* from aqueous solutions containing a mixture of Nd^3+^, Dy^3+^ and La^3+^ at pH 2.5, with an efficiency higher than 90% and at a lanthanide concentration of 0.5 ppm (Minoda et al. 2015). The authors also showed that REEs accumulated inside cells, i.e. they were not only adsorbed onto the cell wall. *G. sulphuraria* accumulated significant levels of Ce, Nd, La and Y from red mud, a byproduct of alumina production (Náhlík et al. 2023), and REEs from waste luminophores (Singh et al. 2023). Another red alga *Galdieria phlegrea* was used to test bioaccumulation of REEs from luminophores from fluorescence lamps and energy saving light bulbs added into the medium in the form of a powder. Algal cells were cultured mixotrophically in a liquid medium with the addition of glycerol as a source of carbon. *G. phlegrea* could grow in the presence of luminophores and accumulate REEs (Čížková et al. 2021). The cyanobacterium *Anabaena* accumulated dissolved Eu, Sm and Nd and formed Eu particles inside its cell (Fischer et al. 2019). Successful biosorbents of La are also immobilized microalgal cells *Ankistrodesmus* sp. and *Golenkinia* sp. (Correa et al. 2016). Recovery of REEs from red mud, was successfully tested in three species of green microalgae *D. quadricauda*, *C. reindhardtii* and *Parachlorella kessleri*. The best growing species was *D. quadricauda* (2.71 cell doublings /day), which accumulated REEs to the highest level (27.3 mg/kg/day), compared with *C. reinhardtii* and *P. kessleri* (Čížková et al. 2019a). As a promising accumulator of REEs, *N. oculata* was studied, accumulating up to 83% of Ce (Wu et al. 2022). Algae and cyanobacteria efficient in the recovery of REEs are summarized in Table 2.

**Table 2:** Algae and cyanobacteria efficient in REE recovery

REE	Alga	Mechanism	Conditions	References
Nd	*Ankistrodesmus gracilis* *Ankistrodesmus densus* *Monoraphidium* sp*.* *Chlorella minutissima* *Euglena gracilis* *Chlamydomonas reinhardtii* *Arthronema africanum* *Calothrix brevissima* *Chlorella sorokiniana* *Euglena mutabilis* *Euglena stellata* *Euglena viridis* *Galdieria sulphuraria* *Lyngbya taylori* *Nostoc ellipsosporum* *Nostoc punctforme* *Porphyridium purpureum* *Prymnesium saltans* *Taselmis chuii* *Arthrospira platensis* *Messastrum gracilis*	Biosorption Biosorption Biosorption Biosorption Bioaccumulation Biosorption Biosorption Biosorption Biosorption Biosorption Biosorption Biosorption Biosorption Biosorption Biosorption Biosorption Biosorption Biosorption Biosorption Biosorption Biosorption	batch, 1d, pH1.5 batch, 1d, pH1.5 batch, 1d, pH1.5 batch, 1d, pH1.5 batch, 30 min batch, 3 h batch, 3 h batch, 3 h batch, 3 h batch, 3 h batch, 3 h batch, 3 h batch, 3 h batch, 3 h batch, 3 h batch, 3 h batch, 3 h batch, 3 h batch, 3 h batch, 3 h batch, 3 h	Palmieri et al. (2000) Guo et al. (2000) Shen et al. (2002) Heilmann et al. (2015)
La, Ce	*Chaetoeros mulleri* *Pavlova lutheri* *Tetraselmis chuii* *Nannochloropsis* spp*.* *Microcystis* spp.	Biosorption Biosorption Biosorption Biosorption Biosorption	batch, 10d, 35 °CC, 26 °CC batch, 10d, 35 °CC, 26 °CC batch, 10d, 35 °CC, 26 °CC batch, 10d, 35 °CC, 26 °CC batch, 4d, 28 °CC, pH8, 2000 lx	Richards and Mullins (2013) Zhou et al. (2004)
La, Eu; Nd	*Chlorella vulgaris*	Biosorption Biosorption Biosorption Biosorption	batch, 20 min, pH3 batch, 20 min, pH4, pH5 batch, 90 min, 21 °CC, 35 °CC, 50 °CC, pH5 batch, RT, 300 µmol/s/m^2^	Heidelmann et al. (2017) Ozaki et al. (2003) Kücüker et al. (2017) Tunali and Yenigun (2021)
Er, Yb; La, Nd, Eu, Gd; Sm, Pr	*Sargassum* sp.	Biosorption Biosorption	batch, 30–40 min, 20 °CC, pH5 batch, 20 °CC, 30 °CC, pH5	Palmieri et al. (2001) Oliveira and Garcia (2009) Oliveira et al. (2012) Oliveira et al. (2011)
La, Eu, Yb	*Sargassum polycystum* *Sargassum hemiphylum* *Schizymenia dubyi*	Biosorption Biosorption Biosorption	batch, 1d, pH3, pH4.5 batch, 1d, pH3, pH4.5 batch, 1d, pH4	Diniz and Volesky (2005, 2006) Kano (2013)
La, Ce, Eu, Yb	*Turbinaria conoides*	Biosorption	batch, 50 min, 6 h, pH4.9	Vijayaraghavan et al. (2010, 2011)
La, Yb	*Ulva pertusa*	Biosorption	batch, 1d, pH4	Kano (2013)
Y, Eu, La, Ce; La, Nd, Dy	*Ulva* sp*.*	Biosorption	batch batch	Viana et al (2023) Manikandan and Lens (2022)
La	*Sargassum fluitans* *Chloroidium saccharophilum* *Stichococcus bacillaris* *Desmodesmus multivariabilis* *Chlorella vulgaris* *Scenedesmus acuminutus* *Chlamydomonas reinhardtii* *Ankistrodesmus* sp*.* *Golenkinia* sp*.* *Ulva innatifida* *Sargassum hemiphyllum*	Biosorption Biosorption Biosorption Biosorption Biosorption Biosorption Biosorption Biosorption Biosorption Bioaccumulation Bioaccumulation	batch, 45 min, pH4, pH5 batch, 5 h, pH6 batch, 5 h, pH6 batch, 6 h, pH6 batch, 5 h, pH6 batch, 5 h, pH6 batch, 5 h, pH6 batch, 8 h, 23 °CC, pH7.5 batch, 8 h, 23 °CC, pH7.5 batch, 1.25d, 15 °CC, pH3 batch, 1.25d, 15 °CC, pH3	Palmieri et al. (2001) Birungi and Chirwa (2013) Birungi and Chirwa (2014) Correa et al. (2016) Sakamoto et al. (2008)
Eu	*Acutodesmus acuminutus*	Biosorption	batch, 40 °CC, pH4, pH7	Furuhashi et al. (2019)
Nd, Eu	*Chlorella brevissima* *Chlorella kessleri* *Calothrix brevissima*	Biosorption Biosorption Biosorption	batch, RT, 25 °CC, 60, 80, 100 µmol/s/m^2^ batch, RT, 25 °CC, 60, 80, 100 µmol/s/m^2^ batch, RT, 25 °CC, 60, 80, 100 µmol/s/m^2^	Heilmann et al. (2021)
Pr	*Turbinaria conoides* *Sargassum wightii*	Biosorption Biosorption	batch, 1 h, pH5 batch, 1 h, pH5	Vijayaraghava and Jegan (2015)
La, Nd, Dy; Y, Sm; Ce, Eu, Tb	*Galdieria sulphuraria*	Bioaccumulation Bioaccumulation Bioaccumulation	batch, 0.5d, 42 °CC, pH1.5 batch, 45 °CC, pH3 batch, 1d, 37 °CC, pH2.5–5.5, 50 µmol/s/m^2^	Minoda et al. (2015) Sun et al. (2022) Iovinella et al. (2022)
Sc, REE	*Posidonia oceanica*	Biosorption	batch, 1d, 20 °CC, pH5	Ramasamy et al. (2019)
La, Ce	*Cystoseira indica*	Biosorption	batch, 8 h, 55 °CC, pH5–5.5	Keshtkar et al. (2018)
Ce	*Arthrospira* sp*.*	Biosorption	batch, pH5–5.5, 300 µmol/s/m^2^	Sadovsky et al. (2016)
Y, La, Ce, Pr, Nd, Eu, Gd, Tb, Dy	*Ulva lactuca* *Ulva intestinalis* *Fucus spiralis* *Fucus vesiculosus* *Gracilaria* sp*.* *Osmundea pinnatifida*	Biosorption Biosorption Biosorption Biosorption Biosorption Biosorption	batch, 3d, 20 °CC, pH8.5 batch, 3d, 20 °CC, pH8.5 batch, 3d, 20 °CC, pH8.5 batch, 3d, 20 °CC, pH8.5 batch, 3d, 20 °CC, pH8.5 batch, 3d, 20 °CC, pH8.5	Pinto et al. (2020)
La, Ce, Nd, Gd	*Desmodesmus quadricauda*	Bioaccumulation	batch, 1d, 30 °CC, pH6.5–7.5, 50–750 µmol/s/m^2^	Řezanka et al. (2016)
Y, Ce, Nd, Eu, La	*Gracilaria gracilis*	Bioaccumulation	batch, 2d, 20 °CC	Jacinto et al. (2018)
Tm	*Turbinaria conoides*	biosorption	batch, 3.5 h, 32 °CC, pH5	Rangabhashiyam and Vijayaraghavan (2019)
Ce, Nd, Tb, La	*Nostoc* sp*.* *Synechococcus elongatus* *Calothrix brevissima* *Desmonostoc muscorum* *Komarekiella*	Biosorption Biosorption Biosorption Biosorption Biosorption	Batch, 23 °CC, 37 °CC, pH8, 300 µmol/s/m^2^ batch, 23 °CC, 37 °CC, pH8, 300 µmol/s/m^2^ batch, 23 °CC, 37 °CC, pH8, 300 µmol/s/m^2^ batch, 23 °CC, 37 °CC, pH8, 300 µmol/s/m^2^ batch, 23 °CC, 37 °CC, pH8, 300 µmol/s/m^2^	Paper et al. (2023)
Eu, Sm, Nd	*Anabaena* sp*.* *Anabaena cylindrica*	Biosorption Biosorption	batch, 12d, 22 °CC, pH6.8 batch, 12d, 22 °CC, pH6.8	Fischer et al. (2019)
REEs from luminophores	*Galdieria sulphuraria* *Galdieria phlegrea*	Bioaccumulation Bioaccumulation	batch, 1d, 40 °CC, pH3, 350 µmol/s/m^2^ batch, 5d, 39 °CC, pH4, 150 µmol/s/m^2^	Singh et al. (2023) Čížková et al. (2021)
REEs from red mud	*Galdieria sulphuraria* *Phormidium *sp. *Oscillatoria *sp. *Lyngbya* sp. *D. quadricauda* *C. reinhardtii* *P. kessleri*	Bioaccumulation Bioaccumulation Bioaccumulation Bioaccumulation Bioaccumulation Bioaccumulation Bioaccumulation	batch, 3d, 40 °CC, pH3, 500 µmol/s/m^2^ batch, 45d batch, 45d batch, 45d batch, 5d, 30 °CC, pH 6.5–7.5, 500 µmol/s/m^2^ batch, 5d, 30 °CC, pH6.5–7.5, 500 µmol/s/m^2^ batch, 5d, 30 °CC, pH 6.5–7.5, 500 µmol/s/m^2^	Náhlík et al. (2023) Dubey and Dubey (2011) Čížková et al. (2019a)
REEs from ore	*Phormidium*	Biosorption	batch, 1–120 min, RT, pH 1–8	Kim et al. (2011)

## Conclusion

The global significance of REEs underscores the potential for substantial benefits through research progress in their recovery. Advancements in REE recovery methods not only contribute to a more secure and diversified supply chain but also alleviate dependence on limited sources. Additionally, enhanced recovery techniques can mitigate environmental impacts associated with conventional extraction, promoting sustainability and responsible utilization of resources. Progress in REE recovery research holds the promise of fostering technological innovation, economic growth, and environmental stewardship on a global scale.

However, despite the promise of microbial recovery methods, challenges remain, including optimizing the efficiency of REE recovery, scaling up laboratory processes for industrial applications, and understanding the ecological impact of introducing engineered microorganisms into natural environments. To overcome all these challenges, research in this field needs to focus on improving the efficiency, cost-effectiveness and environmental sustainability of microbial recovery methods for REEs, including exploring mechanisms and molecular regulation within cells during bio-recovery.

A multidisciplinary approach is crucial for addressing the challenges associated with the recovery of REEs. A comprehensive strategy that integrates expertises from various fields such as geology, chemistry, engineering, and environmental science is essential. Additionally, economists, policymakers, and industry experts are important in creating a supportive framework that incentivizes responsible REE recovery. Ultimately, a multidisciplinary strategy will not only enhance the efficiency of the recovery process but will also promote sustainable practices, contributing to the responsible utilization of these critical resources.

## Data Availability

No datasets were generated or analysed during the current study.
